# Effect of continuous sugarcane bagasse-derived biochar application on rainfed cotton (*Gossypium hirsutum* L.) growth, yield and lint quality in the humid Mississippi delta

**DOI:** 10.1038/s41598-023-37820-8

**Published:** 2023-07-06

**Authors:** Srinivasa R. Pinnamaneni, Isabel Lima, Stephanie A. Boone, Saseendran S. Anapalli, Krishna N. Reddy

**Affiliations:** 1 Crop Production Systems Research Unit, USDA-ARS, P.O. Box 350, Stoneville, MS 38776 USA; 2 Southern Regional Research Center, Commodity Utilization Research, USDA-ARS, 1100 Robert E. Lee Blvd., New Orleans, LA 70124 USA; 3 Department of Environmental Science, University of Arizona, Tucson, AZ 85745 USA; 4 Sustainable Water Management Research Unit, USDA-ARS, P.O. Box 327, Stoneville, MS 38776 USA; 5 Western Colorado Research Center-Grand Valley, Colorado State University, Fruita, CO 81521 USA

**Keywords:** Plant sciences, Biological techniques

## Abstract

Optimizing soil health through soil amendments is a promising strategy for enhancing rainwater efficiency for stabilizing crop production. Biochar, obtained by torrefaction of sugarcane bagasse, a byproduct from sugar mills, has a high potential for its use as a soil amendment, which can boost crop yields, but needs further field trials for its adoption in farming systems. A field study was conducted during 2019–2021 at Stoneville, Mississippi, to assess rainfed cotton (*Gossypium hirsutum* L.) production under four biochar levels (0, 10, 20, and 40 t ha^−1^) on Dundee silt loam soil. The effects of biochar on cotton growth and lint yield and quality were examined. Biochar levels had no significant impact on cotton lint and seed yield for the first two years. Still, in the third year, a significant increase in lint yield by 13 and 21.7% was recorded at 20 and 40 t ha^−1^ biochar levels, respectively. In the third year, lint yields were 1523, 1586, 1721, and 1854 kg ha^−1^ at 0, 10, 20 and 40 t ha^−1^ biochar levels, respectively. Similarly, cotton seed yield increased by 10.8% and 13.4% in 20 and 40 t ha^−1^ biochar plots. This study demonstrated that successive biochar applications at 20 or 40 t ha^−1^ can enhance cotton lint and seed yields under rainfed conditions. These improved yields with biochar did not produce increased net returns due to the increased production costs. Many lint quality parameters were unaffected except for micronaire, fiber strength and fiber length. However, potential long-term benefits of enhanced cotton production from biochar application beyond the length of the study merit further investigation. Additionally, biochar application is more relevant when accrued carbon credits through carbon sequestration outweigh the increased production costs due to biochar application.

## Introduction

Cotton is the most important cash crop yielding natural fiber worldwide, with an acreage of 34 million ha in 85 countries^1^. In the Mississippi, USA, cotton is grown in over 0.18 million ha with an estimated production of 0.89 million bales^2^, where about 40% of cotton area is rainfed^3^. The dairy industry values whole cotton seed and seed meal due to their high protein (35%) and oil (30%) content^4^. Cotton cultivation is technology intensive with seed costs, processing technology fees, high nitrogen fertilizer needs, and irrigation costs^5,6^. Conservation agricultural practices like no-till, cover cropping, rotation with a legume like soybean, and soil amendments use, are expected to increase the sustainability of cotton production compared with conventional cotton production in Mississippi^7–9^.

Biochar used in this study is a pyrogenic carbon-rich porous recalcitrant material produced by pyrolysis of sugarcane bagasse at low temperature (approximately 300 °C) under an inert atmosphere, by American Biocarbon LLC, White Castle, Louisiana, USA. Biochar has attracted researchers’ interest mainly due to its long-term soil carbon (C) sequestration potential, role in greenhouse gas mitigation, phytoremediation, and ability to improve soil fertility^10–13^. Biochar can be made from various biomass materials/residues such as wood waste, crop and refinery residues, animal manures, and municipal wastes. Biochar derived from crop residues can be a sustainable management option as often crop residues are underutilized or burnt and removed from agricultural fields^14^. The beneficial effects of biochar application on crop yield, soil fertility, and health vary with the type and amount of biochar applied, fertilizer use, soil properties, climate, and cumulative effects from repetitive applications^11,14–16^. Gaskin et al. (2008) reported that applying peanut hull and pine chip biochar for two years improved soil organic carbon (SOC) and N content in Ultisols in the southeastern United States^17^. However, pine chip biochar addition decreased corn grain yields linearly with application rate (0, 11, and 22 t ha^−1^). In comparison, peanut hull biochar decreased grain yields at 22 t ha^−1^ application rate, and overall responses were smaller than expected^18^. Another study^15^ (demonstrated a single dose application of 20 t ha^−1^ on savannas Oxisol of Colombia improved corn grain yield by 28, 30, and 140% from control in the second, third, and fourth years of study. However, no impact on grain yield was observed in the first year of the study^15^. Application of corn straw biochar rates between 5 and 20 t ha^−1^ for three successive years in cotton grown on Inceptisol in China resulted in 8.0–15.8%, 9.3–13.9%, and 9.2–21.9% lint yield enhancement. Wheat straw biochar at 10–40 t ha^−1^ on rice grown on hydrologic Stagnic Anthrosol in China resulted in a 10% yield rise for the first year and 9.5–29% in the second year^19^. In Georgia, the yield or quality responses in drip-irrigated cotton, corn, or peanuts were insignificant, with biochar rates of 0, 22.4, 44.8, 89.6, and 134.4 t ha^−1^^20^. A global meta-analysis of biochar studies revealed a highly variable impact of biochar on crop yield, more evident in large plot trials. This is probably due to various factors ranging from feedstock used in biochar production, application rate, pyrolysis conditions (temperature and residence time), soil type and fertility, soil-crop-water management, and climate^21^.

Each year, an estimated 0.6 million tons of sugarcane bagasse is available as a byproduct from sugarcane processing mills in Louisiana, USA^12^. Pelleted biochar is produced by torrefaction of surplus sugarcane bagasse and can be used as a soil amendment to improve soil health parameters such as bulk density, aggregate stability, hydraulic conductivity, nutrient availability, microbial dynamics, etc., in agriculture. Understanding the effect of biochar as a soil amendment on grain yield and quality will significantly influence producer’s adoption rate. No studies are available on the effects of biochar (produced by the torrefaction of sugarcane bagasse) on cotton productivity and lint quality. Hence, a field study was conducted by applying the biochar in the pelleted form to soil under rainfed cotton production at four rates for three consecutive years to assess (1) cotton growth, (2) lint yield and quality, and (3) farm profitability.

## Materials and methods

### Experimental site and design

**Figure 1:**
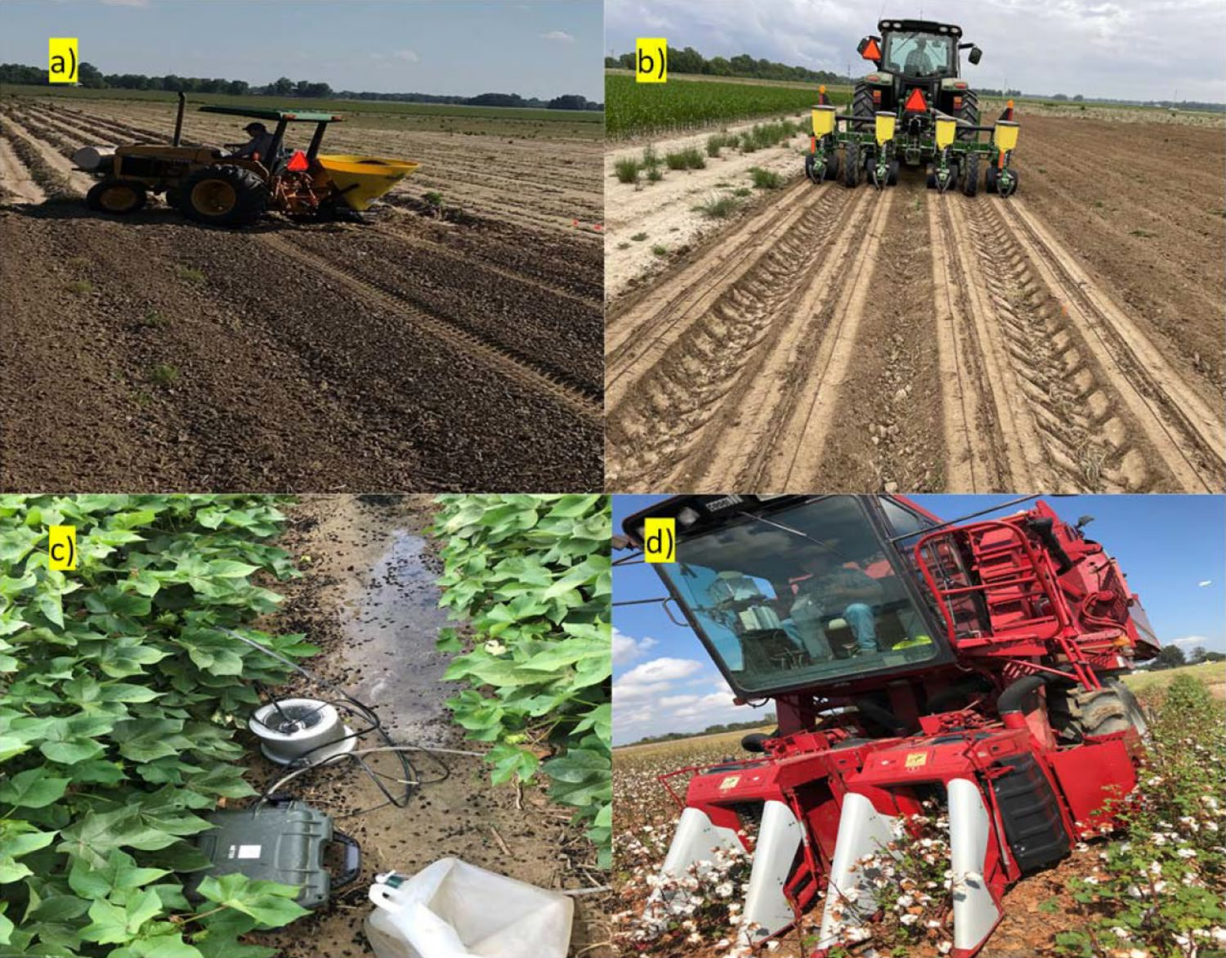
Illustration of (**a**) pelleted biochar application and (**b**) cotton seed planting, (**c**) saturated hydraulic conductivity measurement, (**d**) cotton picking in the experiment conducted at Stoneville, Mississippi, USA.

Field studies were conducted at the USDA-ARS Crop Production Systems Research Unit’s (CPSRU) farm in Stoneville, Mississippi, USA (33°42′N, 90°55′W, elevation: 32 m above mean sea level) during 2019, 2020 and 2021 crop seasons on Dundee silt loam (fine silty, mixed, active, thermic Typic Endoaqualfs) with 0.91% organic matter, 6.8 pH, 0.42% carbon, 0.07% nitrogen, and 1.32 g cm^−3^ bulk density averaged across 60 cm soil depth. The field-saturated hydraulic conductivity (Kfs) of the soil ranged from 0.41 to 1.22 cm h^−1^. The experiment was a randomized complete block design with six replicates of four biochar application rates, (1) 0, (2) 10, (3) 20, and (4) 40 t ha^−1^. These biochar application rates were intentional to try and mimic biochar accumulation and its effects over time at different rates. All biochar rates were randomly assigned in the first year of the study, and then the same application rates were applied to the assigned plots for the remaining two years to assess the impact of continuous use. The biochar application was calibrated using a tractor-mounted forage seed spreader for 2.5 t ha^−1^ for every pass, and 2, 4, 8, and 16 passes were made to apply 5, 10, 20, and 40 t ha^−1^ rates (Fig. 1). Biochar from sugarcane bagasse was produced via torrefaction (low-temperature pyrolysis; approximately 30 min residence time). Torrefied bagasse was then pelletized. The biochar was supplied in a pelleted form (0.25 in diameter pellets) by American Biocarbon CT LLC, White Castle, Louisiana, USA, at USD 50 per ton in 2019. Some of the critical operations are shown in Fig. 1.

### Trial management

Field preparation in the fall consisted of one deep tillage operation to break clay pans and overturn soils, burying crop residue, and killing weeds, followed by a disc-tillage to generate furrows and ridges (102 cm row spacing) for planting cotton seeds. In the spring, glyphosate at 1.12 kg active ingredient (a.i.) ha^−1^ was applied 1–2 weeks before planting cotton to kill existing weeds. A 7300-vacuum planter (John Deere, East Moline, IL) was used to plant cotton at 120,000 seed ha^−1^. The recommended population in Mississippi is 100,000–125,000 plants ha^−1^ for 102 cm row cotton^22^. Actual plant populations were estimated at harvest by counting plants in a 1 m row length at three randomly selected locations in each plot. Recommended preemergence and postemergence herbicide programs were followed to manage weeds. Pre-plant spraying of paraquat at 1.05 kg a.i. ha^−1^ was done for killing existing weeds as needed. Fluometuron at 1.12 kg a.i. ha^−1^, and pendimethalin at 1.12 kg a.i. ha^−1^ was used for preemergence weed control. For postemergence control, glufosinate-ammonium at 0.6 kg a.i. ha^−1^ was applied twice. Escaped weeds were hand-hoed to keep plots weed free. Standard insect control programs for cotton production were applied. Every year, at the V5 stage, 88 kg N ha^−1^ was applied to all the experimental plots as a urea-ammonium nitrate solution (32%). In October 2020, an airplane applied 70 kg ha^−1^ of potassium in the form of potassium sulfate (43% K) after cotton harvest and shredding. Cotton seed variety “Phytogen PHY 430 W3FE” with early maturity, good vigor, broad adaptation, and high yield potential was planted on May 16, 2019, May 14, 2020, and May 18, 2021. Each plot consisted of six rows and was 20 m long. Cotton was defoliated during mid-late September when approximately 65% of the bolls had opened. In mid-September, a mixture of 0.035 kg thidiazuron ha^−1^ and 0.0175 kg diuron ha^−1^ was applied initially. Later, 0.035 kg thidiazuron ha^−1^, 0.0175 kg diuron ha^−1^, and 1.68 kg ethephon ha^−1^ were used one week after the first spray to promote leaf senescence and boll opening.

### Plant sampling and data collection

Plant phenology data were collected periodically from the date of emergence (Vo) till the harvest (C5), characterized as functions of the phenological phases, namely, vegetative (V), formation of flower buds (B), flowering (F) and boll cracking (C)^23^. Leaf area index (LAI) was measured at biweekly intervals using an AccuPAR LP 80 Ceptometer (Decagon Devices, Inc., Pullman, WA, USA). Plant heights were collected at the boll cracking (C5) stage. All plant measurements were replicated at five random locations in the plot. Above-ground biomass was harvested from a 1 m long section of bed from each plot at three areas, avoiding the row ends, to collect data on the number of open bolls and plants. Seed cotton was handpicked at the C5 stage. The harvest index was calculated as a ratio of dry weights of plants and seed cotton. The 1822 Case IH 2-row cotton harvester (CNH Industrial America LLC, WI, USA) was used for harvesting the plots. Seed cotton was ginned on a 10-saw laboratory gin (Continental Eagle, Prattville, AL, USA), and the lint yield and seed yield were calculated per hectare basis. One hundred seeds were counted and weighed to get 100-seed weight.

Precipitation water use efficiency (PWUE) (kg lint mm^−1^ of rainfall) was calculated as:

1 $$PWUE = \left( \frac{Y}{P} \right)$$

where Y is the lint yield in a treatment (with or without biochar) and P is the cotton growing season precipitation (mm) recorded, *Yi* is the lint yield in the biochar applied treatment (kg ha^−1^), and *Yr* is the lint yield in the non-biochar plot (kg ha^−1^). The biochar efficiency index (BEI) was calculated as

2 $$BEI = \left( {\frac{Yi - Yr}{Q}} \right)X 100$$

where Q is the quantity of biochar applied (t ha^−1^), weather data was downloaded from an Agricultural Weather Service, Mississippi State University, Stoneville, Mississippi). The GDD (°C) were calculated using a base temperature (*T base*) of 10 °C and an upper threshold of 30 °C^24^

3 $$GDD = \left( {\frac{Tx + Ty}{2}} \right) - Tbase$$

where (Tx + Ty)/2 < 10 or (Tx + Ty)/2 > 30. GDD = 0.0.; where Tx and Ty are the daily maximum and minimum air temperatures.

### Fiber quality analysis

Ten subsamples were collected after the lint cleaner from each sample for fiber quality analysis; five for High-Volume Instruments (HVI) and five for Advanced Fiber Information Systems (AFIS). All lint samples for HVI were analyzed in the USDA-ARS Cotton Ginning Research Unit (CGRU) in Stoneville, Mississippi. AFIS analysis was performed at Fiber and Biopolymer Research Institute, Texas Tech University, TX. The parameters measured were HVI: micronaire, fiber length, uniformity index, strength, elongation, yellowness, reflectance, upper half mean length (UHML) and AFIS: nep, short fiber content (SFC), upper quartile length (UQL), fineness, maturity ratio, fiber length by number, fiber length by weight and visible foreign matter (VFM).

### Economic analysis

The Mississippi State University’s Department of Agricultural Economics planning budgets were used to compute cotton production costs^5,25,26^. The production cost of cotton was taken directly from the published budget reports. Operational costs included: land preparation, fertilizer, herbicide, seed, planting, spraying, tractor and combine operation, hauling, fuel, and interest on operating capital. Fixed costs include implements, tractors, and combine. The production costs of the biochar treatments were computed by applying biochar cost at USD 50 t^−1^ and application cost. The transportation cost was not included. The crop year average prices for lint were USD 1.57, 1.87, and 2.91 in 2019, 2020, and 2021, respectively, and seed USD 0.18, 0.21, and 0.22 in 2019, 2020, and 2021, respectively were obtained from the National Cotton Council of America’s website (https://www.cotton.org/econ/prices/index.cfm) and used in estimating the returns.

### Biochar properties

Pelleted biochar was evaluated for select physical and chemical properties, including surface area, bulk density, pH, organic carbon, hydrogen/carbon ratio, total nitrogen, potassium and phosphorous, electrical conductivity, liming value, and proximate analysis. Surface area measurements were obtained from nitrogen adsorption isotherms at − 196 °C using a Nova 2200 Surface Area Analyzer (Anton Paar Corp., Boynton Beach, FL). Specific surface areas (BET, Brunner–Emmett–Teller) were taken from adsorption isotherms using the BET equation. Proximate analysis (ASTM method D5142-09) was performed on a Thermogravimetric Analyzer (TGA701, LECO, St. Joseph, MI, USA) to determine moisture, ash, volatile matter (VM), and fixed carbon contents. The remaining properties were obtained from Control Laboratories (Watsonville, CA). Biochar properties are summarized in Table 1.

**Table 1:** Torrefied sugarcane bagasse derived pelleted biochar properties.

Proximate analysis				
Volatile matter (% db)	Fixed carbon (% db)	Ash content (% db)	Surface area (m^2^ g^−1^)	
65.72 ± 0.48	27.79 ± 0.57	6.49 ± 0.14	180	

Bulk density (g cm^−3^)	pH	Organic C % total dry mass	H:C ratio	Electrical conductivity (dS m^−1^)
0.64	5.15	50.8	1.21	0.100

Total N % total dry mass	Total P (mg kg^−1^)	Total K (mg kg^−1^)		
0.30	307	2162		

### Statistical analysis

Data collected on yield responses to treatments were subjected to analysis of variance using PROC MIXED in Statistical Analysis System (JMP version 16.0; SAS Institute Inc., Cary, NC), and treatment means were separated by Tukey HSD test at *P* ≤ 0.05. Biochar rate was considered a fixed effect, and replicates and year were considered random.

## Results

### Seasonal weather

**Figure 2:**
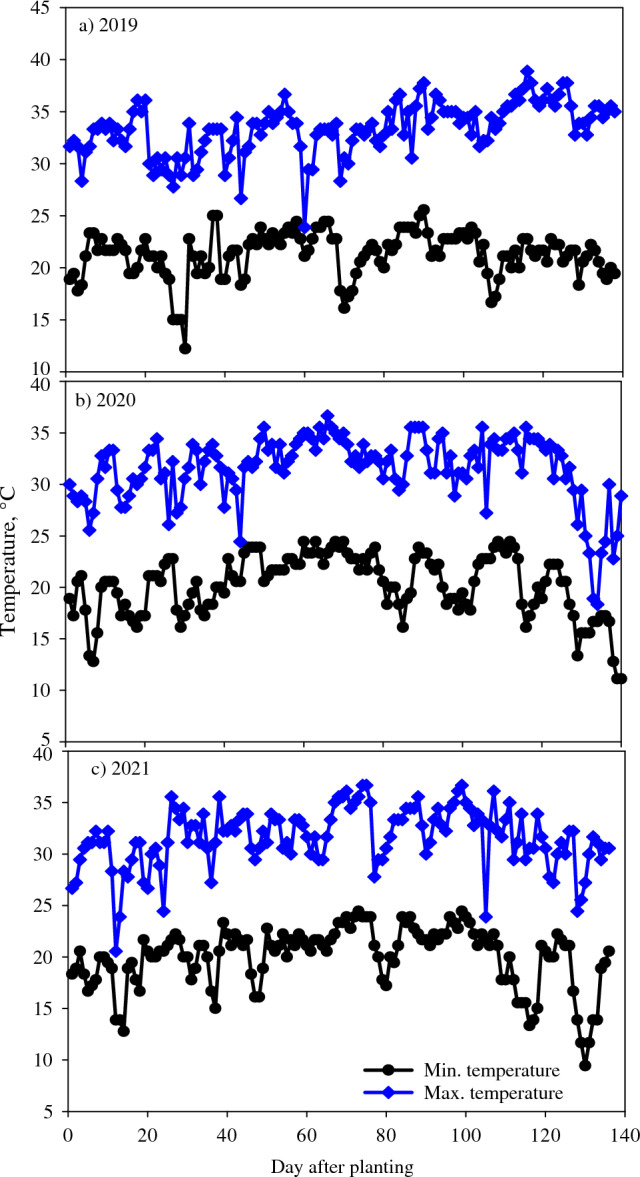
Daily air minimum and maximum temperature, for (**a**) 2019, (**b**) 2020 and (**c**) 2021 cotton growing seasons at Stoneville, Mississippi, USA.

**Figure 3:**
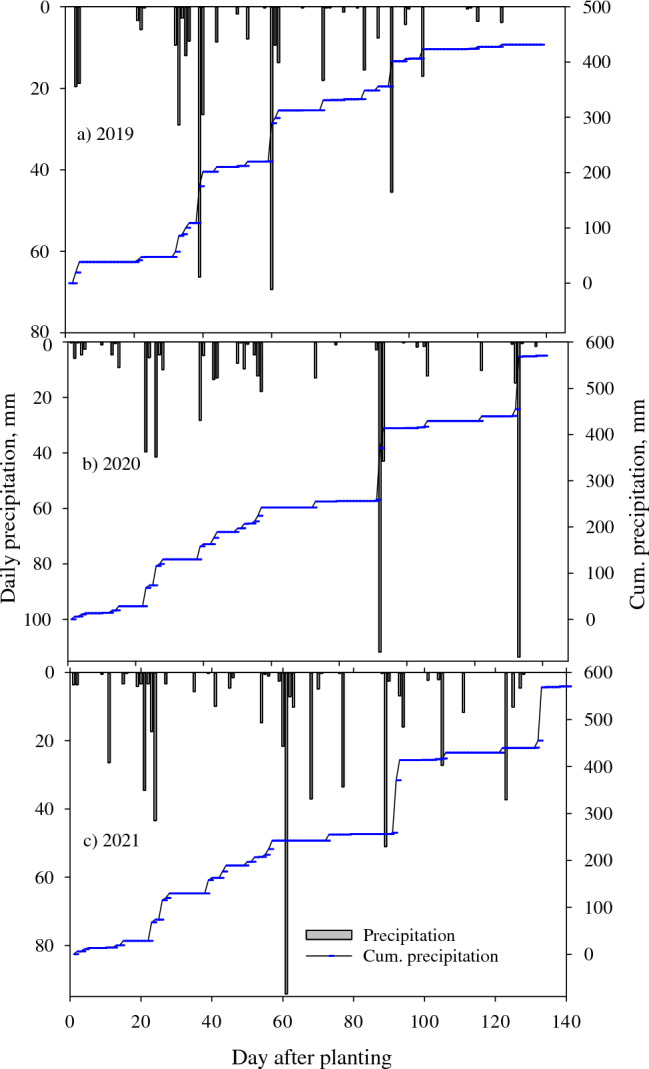
Measured precipitation and cumulative precipitation for (**a**) 2019, (**b**) 2020 and (**c**) 2021 cotton growing seasons at Stoneville, Mississippi, USA.

**Figure 4:**
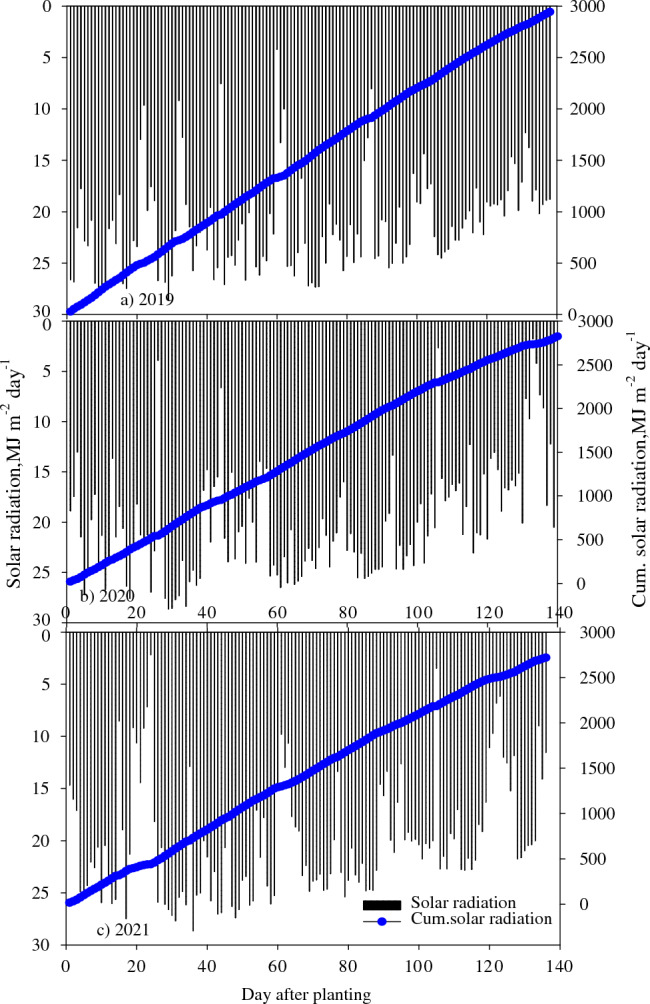
Solar radiation and cumulative solar radiation for (**a**) 2019, (**b**) 2020 and (**c**) 2021 cotton growing seasons at Stoneville, Mississippi, USA.

The measured weather during the three growing seasons (2019, 2020, and 2021) was highly different (Figs. 2, 3, 4). The minimum and maximum temperatures during flowering, boll development, and ball cracking occurred during July–September, ranging from 21.7 to 34.2 °C in 2019, 20.6 to 32.0 °C in 2020, and 20.1 to 32.1 °C in 2021 (Fig. 2). During the same period, measured precipitation differed highly across the seasons, with 221, 400, and 380 mm recorded in 2019, 2020, and 2021, respectively (Fig. 3); the 2019 season received 81 and 72% lower precipitation than the 2020 and 2021 seasons during the cotton reproductive phase. The mean daily solar radiation during July–September was 20.5, 19.5, and 19.4 MJ m^−2^ in 2019, 2020, and 2021, respectively (Fig. 4). The differences in weather during the reproductive phase of cotton boll development have contributed to the significant variation in lint yield and quality parameters across the three seasons (Tables 2–3).

**Table 2:** Cotton lint and seed yield components in response to different levels of biochar in 2019, 2020 and 2021 at Stoneville, Mississippi, USA.

Year	Biochar rate (t ha^−1^)	Plant population ha^−1^	Plant height (cm)	Number of open bolls ha^−1^	Boll weight (g boll^−1^)	Lint yield (kg ha^−1^)	Lint yield over control (%)	100-seed weight (g)	Harvest index	Seed yield (kg ha^−1^)	Seed yield over control (%)
2019	0	78740d	69.8a	541339c	8.3a	1189b		14.7b	0.33b	2946b	
	10	83661c	69.5a	600394b	8.0b	1236a	3.95	14.8b	0.32c	3128a	6.18
	20	93504a	68.4a	531496c	7.6c	1184b	-0.42	15.0a	0.34a	2985b	1.32
	40	88583b	70.5a	610236a	7.9b	1195b	0.50	14.9a	0.33b	3147a	6.82
2020	0	78898b	76.9c	629921c	8.2b	1644b		14.8b	0.36b	3780b	
	10	88582a	78.5c	708661b	8.5a	1703a	3.59	14.8b	0.36b	3911a	3.30
	20	78493b	86.7b	688976b	8.2b	1696a	3.16	15.2a	0.35c	3821a	0.92
	40	86498a	91.6a	748031a	8.3b	1718a	4.50	15.1a	0.37a	3924a	3.65
2021	0	83661b	68.9b	639764c	7.4d	1523c		15.1c	0.36c	3587b	
	10	83661b	72.3b	669291c	7.9c	1586c	4.14	15.2b	0.34d	3652b	1.81
	20	88583b	75.6b	738189b	8.2b	1721b	13.00	15.3a	0.37b	3976a	10.84
	40	98425a	88.5a	816929a	8.5a	1854a	21.73	15.3a	0.39a	4068a	13.41
Source of variance											
Year		**	*	**	*	**		*		**	
Treatment		*	ns	ns	*	**		*		*	
Year × treatment			ns	ns	ns	ns		ns		ns	

*ns* non-significant.

*Significantly different at *P* ≤ 0.05 level.

**Significantly different at *P* ≤ 0.01 level. Means followed by the same letters are not significantly different according to Tukeys HSD test at 5% significance level in the same year.

**Table 3:** Cotton lint quality parameters in response to different levels of biochar in 2019, 2020 and 2021 at Stoneville, Mississippi, USA.

Year	Biochar rate (t ha^−1^)	Micronaire	Uniformity (%)	UHML (mm)	Strength /(kN m kg^−1^)	Reflectance (Rd)	Elongation rate (%)	Yellowness (+ b)	Nep/(cnt/g)	Fiber length_n_ (mm)	SFC_n_ (%)	VFM (%)	UQL (w)	Maturity ratio	Fineness
2019	0	5.21a	81.30a	1.18a	33.25a	74.72a	5.96a	6.90b	93b	1.41a	21.50b	5.77b	1.25a	0.97a	179a
	10	4.95b	81.05a	1.15b	33.67a	74.85a	6.02a	7.15a	64c	1.42c	18.40c	5.20b	1.27a	0.96a	177b
	20	5.20a	81.27a	1.18a	32.30b	72.92	5.97a	7.07a	201a	1.36b	24.80a	6.94a	1.20a	0.96a	179a
	40	5.15a	80.55b	1.15b	32.70b	73.60	5.90a	6.97b	110b	1.36b	21.60b	3.77c	1.19a	0.96a	180a
2020	0	4.50c	83.70c	1.09b	31.85c	72.25a	5.93a	8.88a	87a	1.31c	21.38a	6.10a	1.17b	0.92a	178a
	10	4.69a	84.60a	1.14a	33.88a	72.85a	5.73c	8.83a	87a	1.37a	19.95b	4.55c	1.22a	0.92a	172b
	20	4.61b	83.98b	1.14a	31.65c	72.38a	5.83b	8.70a	87a	1.36a	20.90b	6.26a	1.22a	0.92a	177a
	40	4.75a	84.28a	1.14a	32.13b	72.55a	5.95a	8.85a	85a	1.34b	21.70a	5.79b	1.20a	0.92a	177a
2021	0	4.39c	81.97c	1.08b	31.50b	72.90b	6.2b	8.28a	154c	1.08a	26.40b	4.44b	1.14a	0.89b	169a
	10	4.46b	82.70a	1.11a	31.87a	72.97b	6.15b	7.83b	176b	1.11a	28.05a	3.92c	1.14a	0.91a	171a
	20	4.47b	82.32b	1.09a	31.90a	71.87c	6.25b	7.98b	182a	1.09a	26.68b	5.61a	1.15a	0.89b	168a
	40	4.56a	82.95a	1.11a	31.90a	73.12a	6.40a	7.53c	158c	1.11a	24.30c	4.93b	1.17a	0.88b	169a
Source of variance															
Year		**	*	**	*	*	*	*	**	**	*	**	*	**	ns
Treatment		*	*	*	*	ns	*	*	*	*	ns	ns	*	*	ns
Year × treatment		ns	ns	ns	ns	ns	ns	ns	*	ns	ns	ns	ns	ns	ns

*ns* non-significant, *Cnt* count, *VFC* visible foreign matter, *UHML* upper half mean length, *UQL* upper quartile length, *SFC* short fiber length

*Significantly different at *P* ≤ 0.05 level.

**Significantly different at *P* ≤ 0.01 level. Means followed by the same letters are not significantly different according to Tukeys HSD test at 5% significance level in the same year.

### Crop phenology

**Figure 5:**
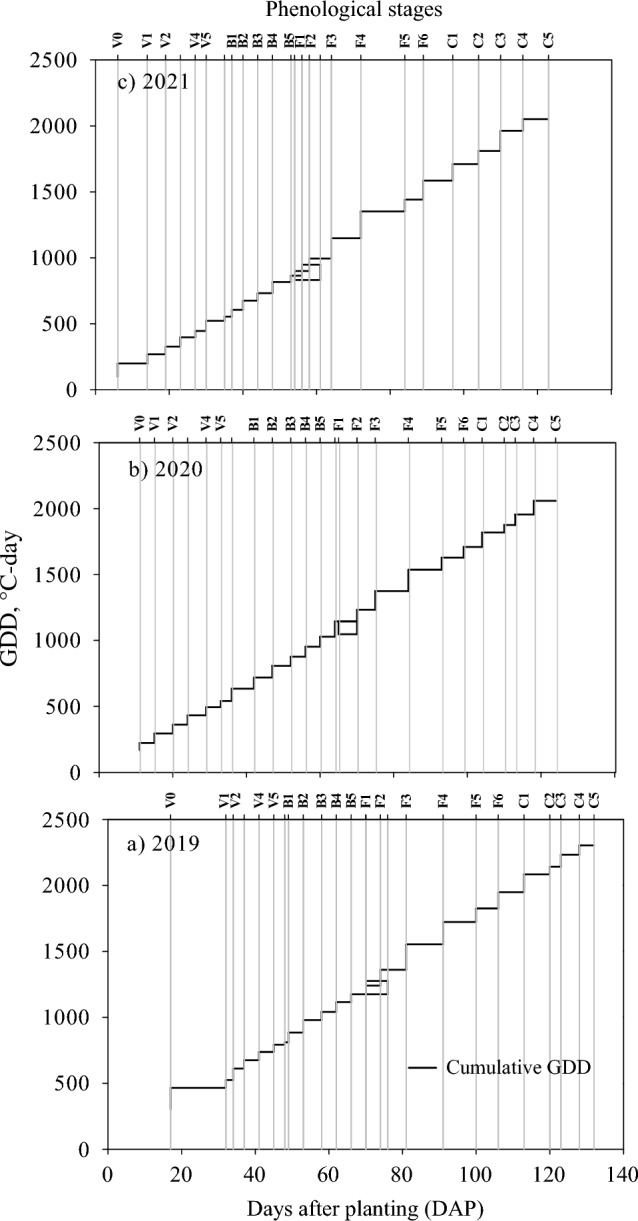
Illustration of crop phenology and accumulated growing degree days in (**a**) 2019, (**b**) 2020 and (**c**) 2021 cotton growing seasons at Stoneville, Mississippi, USA.

The association of different phenological stages with growing degree days is presented in Fig. 5. The high differences in accumulated GDD between Vo and V_1_ are attributed to heavy precipitation of 38.4 mm one day after planting in 2019, and the 2020 and 2021 seasons received 13 and 7 mm, respectively, during the first week. The heavy rain on clay loam soil led to hypoxia for a few days and delayed germination in 2019^6^. Transitioning from the vegetative to the reproductive phase required 792, 541, and 522 GDD in 2019, 2020, and 2021, respectively. The GDD required for the first sympodial flower anthesis were 1175, 1047 and 900 in 2019, 2020 and 2021, respectively. The first cracked boll on the fifth sympodium took 2303, 2058, and 2052 GDD in the three respective cotton growing seasons in all the biochar treatments except for 40 t ha^−1^ biochar level plots which took an additional 5–7 days in each of the three years of study.

### Leaf area index (LAI)

**Figure 6:**
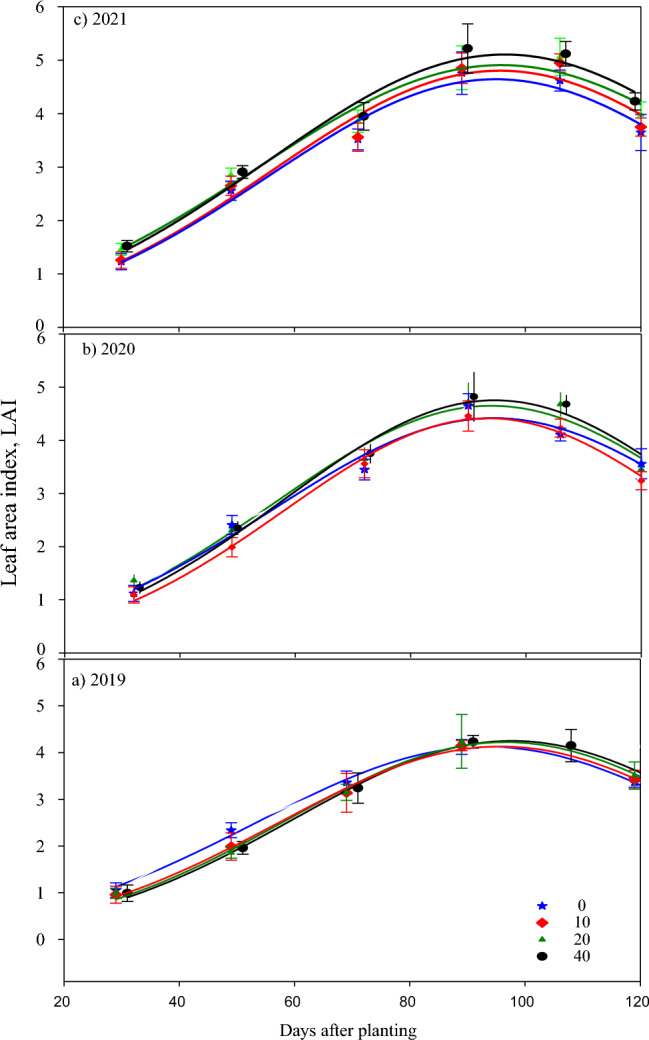
Cotton leaf area index (LAI) during the crop growing seasons in (**a**) 2019, (**b**) 2020 and (**c**) 2021 at different levels of biochar application 0, 10, 20 and 40 t ha^−1^ in Stoneville, Mississippi, USA.

The LAI was measured at biweekly intervals in all three seasons till defoliation, and it is shown in Fig. 6. There appears to be no difference between the treatments for canopy coverage initially, but the high rate of biochar has positively impacted the LAI in all the growing seasons at boll opening. The highest LAI was observed 90 days after planting in all treatments. The 20 and 40 t ha^−1^ biochar treatments recorded over 5 LAI in 2021, while in 2019 and 2020, the highest LAI of 4.2 and 4.9, respectively, was recorded in plants with 40 t biochar ha^−1^. The differences in LAI were negligible among the treatments even during post-flowering in 2019, while the 20 and 40 t ha^−1^ biochar applied plots recorded higher LAI than the other treatments during post-flowering in the 2020 and 2021 seasons.

### Biochar effects on crop agronomy and yield

Some yield components contributing to lint and seed yield, such as plant population ha^−1^, number of open bolls ha^−1^, boll weight, and 100-seed weight, were significantly affected by year and biochar application rates, but the interactions with years were not significant for all the traits (Table 2). The differences were more pronounced in years 2 and 3 due to repeated biochar applications on the same plots. In two out of the three years, high biochar rate applied plots (20 and 40 t ha^−1^) recorded a 15.6% and 8.7% higher population per ha in 2019 and 2021, respectively. In biochar-applied plots, the plant height at the C5 stage was significantly higher (15–19%) in the second and third years (2020 and 2021). In high biochar rate plots, the number of open bolls per ha was significantly higher by 14% and 21.5% in 2020 and 2021, respectively. However, no clear trend was available 2019 for open bolls per ha. Single open boll weight was higher in the control plot for the first year, while high biochar applied plots recorded significantly higher boll weight only in the third year of study. The differences for this trait were non-significant in the second year of study, as the average boll weight across the treatments was higher than in the other two years. The 100-seed weight ranged from 14.8–15.0 g in the first year, and the differences were insignificant. At the same time, the 20 and 40 t ha^−1^ biochar plots recorded significantly higher 100-seed weights ranging from 15.1 to 15.3 g in the following two years (Table 2). The seed yield was significantly higher in biochar-applied plots over the control except for the first year. In 2021, the seed yield was 10.8% and 13.4% higher in 20 and 40 t ha^−1^ biochar-treated plots (Table 2). The lint yield ha^−1^ ranged from 1184–1236 kg ha^−1^, 1644–1718 kg ha^−1^, and 1523–1854 kg ha^−1^ in 2019, 2020, and 2021, respectively. The high-rate biochar application improved lint yields by 3.8% and 17.3% in 2020 and 2021.

### Biochar effects on lint quality

The ANOVA showed significant effects of biochar rate and year for all the HVI and AFIS parameters, except for fineness and upper quartile length (UQL), while their interaction effects were non-significant (Table 3). The highest micronaire was recorded by the control plot (5.21) in 2019, while 40 t ha^−1^ biochar applied plots exhibited the highest micronaire (4.56) in 2021. Except for the first year, biochar-applied cotton recorded significantly higher lint uniformity and strength. Reflectance was highest for the control plots in the first year, while no clear trend was apparent for the other two years. Elongation rate (%) and fiber length (mm) were significantly higher in biochar-amended plots in the second and third years of the study. A clear trend was not observed for yellowness, short fiber content by number (SFCn, %), visible foreign matter (VFM, %), and nep count in any of the years. Only yellowness increased linearly with the biochar application rate in 2021. Neps indicate defects in cotton fiber; this is the only trait observed to be affected by year X treatment interaction. Fiber size and quantity are commonly considered while adjusting the processing machinery to decrease or remove mechanical neps formation. Generally, 0.5 g of the cotton fiber samples are used for estimating the nep count. Maturity ratio, an indicator of matured fibers, ranged between 0.96–0.97 and 0.89–0.91 in 2019 and 2021, respectively, while it was the same for all the treatments in 2020. Based on the three years of data, weather parameters appeared to play a more significant role.

### PWUE and BEI at different biochar application rates

The PWUE varied among treatments and years, with a non-significant biochar rate × year interaction (Table 4). The PWUE ranged from 2.74 to 2.86 kg m^−3^, 2.88 to 3.01 kg m^−3^, and 2.67 to 3.25 kg m^−3^ in 2019, 2020, and 2021, respectively. The PWUE increased with increased biochar application rates only in the third year, while no clear trends were observed in the first two years. The 40 t ha^−1^ application rate exhibited the highest PWUE of 3.01 kg m^−3^ in 2020 and 3.25 kg m^−3^ in 2021. The BEI, a measure of response from a ton of biochar applied, recorded significant differences among biochar levels and years, with a non-significant interaction among them (Table 4). The BEI ranged from − 25 to 470; 185–590 and 630–990 in 2019, 2020, and 2021, respectively. The biochar rate of 10 t ha^−1^ recorded the highest BEI of 470 and 590 in 2019 and 2020, respectively, while 20 t ha^−1^ treatment recorded a high in 2021. Compared to the first year, the higher biochar application rates of 10 and 20 t ha^−1^ resulted in a higher index. In the third year of study, the BEI was similar in 20 t ha^−1^ (990) and 40 t ha^−1^ (828) biochar application rates. It appears that the BEI is increasing with the increase in application rate in the second and third years (2020 and 2021), unlike the 2019 response.

**Table 4:** Precipitation water use efficiency (PWUE) at different biochar application rates in cotton during 2019, 2020 and 2021 seasons, respectively, at Stoneville, MS.

Year	Biochar rate (t ha^−1^)	Lint yield (Kg ha^−1^)	Precipitation (mm)	PWUE (kg m^−3)^	Biochar efficiency index
2019	0	1189	432	2.75b	
	10	1236	432	2.86a	470
	20	1184	432	2.74b	− 25
	40	1195	432	2.76b	15
2020	0	1644	570	2.88b	
	10	1703	570	2.98a	590
	20	1696	570	2.97a	260
	40	1718	570	3.01a	185
2021	0	1523	569	2.67d	
	10	1586	569	2.78c	630
	20	1721	569	3.02b	990
	40	1854	569	3.25a	828
Source of variance					
Year			*		
Treatment			*		
Year × treatment			ns		

*PWUE* precipitation water use efficiency, *BEI* biochar efficiency index, *ns* non-significant.

*Significantly different at *P* ≤ 0.05 level.

Means followed by the same letters are not significantly different according to Tukeys HSD test at 5% significance level in the same year.

### Economics of biochar application

The expected profits from the sale of lint and cotton seed after meeting the production costs and biochar application costs are shown in Table 5. The total revenue per ha varied between USD 2384–2490, 3889–4057, and 5392–6485 during 2019, 2020, and 2021, respectively. The higher range in 2021 is attributable to the enhanced productivity of biochar-amended cotton and high prevailing market lint prices. During the first year of biochar application, only the non-biochar plot returned a profit of USD 265 ha^−1^ as the benefits of biochar addition were not enough to overcome the cost. The losses were proportional to the biochar application levels: USD 143, 750, and 1704 losses accrued in plots applied with 10, 20, and 40 t ha^−1^ biochar. In 2020, the returns from 0, 10 and 20 t ha^−1^ biochar application rates resulted in USD 1787, 1409 and 877 profits, while the highest biochar applied cotton accrued a loss of USD 60. In the 2021 season, all the treatments recorded profits in decreasing order with increased rates of biochar application, that is, USD 3325, 3021, 3001, and 2412 for 0, 10, 20, and 40 t ha^−1^ biochar application rates, respectively.

**Table 5:** Effects of biochar application on cotton profitability in 2019, 2020 and 2021 seasons, respectively, at Stoneville, Mississippi, USA.

Year	Biochar rate (t ha^−1^)	Revenue from lint sale (USD)	Revenue from seed sale (USD)	Total revenue (USD ha^−1^)	Total production cost (USD ha^−1^)	Expected profits (USD ha^−1^)
2019	0	1862	523	2384	2119	265a
	10	1935	555	2490	2633	(143)b
	20	1854	530	2384	3133	(750)c
	40	1871	559	2430	4133	(1704)d
2020	0	3079	810	3889	2102	1787a
	10	3190	836	4026	2617	1409b
	20	3177	817	3994	3117	877c
	40	3218	839	4057	4117	(60)d
2021	0	4435	957	5392	2058	3335a
	10	4619	974	5593	2572	3021b
	20	5012	1061	6073	3072	3001b
	40	5399	1085	6485	4072	2412c

*Profits given within parenthesis show a net loss, *USD* United States Dollar, ≠ numbers within a column followed by the same letter are not significantly different at *P* ≤ 0.05.

## Discussion

Many studies have demonstrated that biochar addition to soil can significantly enhance crop growth, development, and grain yield^10,11,14,19,27^. In general, straw, plant residues, and manure-based biochar give higher crop yields in infertile and acidic soils, partly owing to their greater soil reclamation effect and nutrient levels. In this three-year study, biochar application enhanced cotton lint yields in the second and third year by 3.2–4.5% and 4.1–21.7%, respectively. In the first year, biochar application at various levels produced mixed outcomes in its impacts on lint yields. Enhanced lint yield of 3.9% was observed only at 10 t ha^−1^ level, while 20 and 40 t ha^−1^ treatments had no effect. Successive application of corn straw biochar at 0, 5, 10, and 20 t ha^−1^ to cotton in an Inceptisol in China resulted between 8.0–15.8, 9.3–13.9, and 9.2–21.9% increase in lint yields, respectively. This was attributed to reduced nitrate leaching and increased total nitrogen (N), soil organic carbon, and plant available K in the soil at 20 cm depth in the second and third season of the study^10^. As biochar is recalcitrant, continuous application of biochar resulted in increased soil organic carbon in loamy soils^28^. Onetime application of hardwood biochar at 0, 22.4, 44.8, 89.6, and 134.4 t ha^−1^ did not affect the cotton lint yield or its quality when grown under rainfed or sprinkler irrigation^20,29^. In another analogous study in similar soils of MS using continuous application of poultry litter, resulted in only marginal gain in cotton lint yield. Hence, successive applications of biochar were made. The lack of response of biochar in the first year, in this study, is akin to the earlier report of Major (2010) and the subsequent positive impact on cotton yield is similar to the earlier reports^10,11^. The lack of response in the first year in this study is possibly due, in part, to the pelleted nature of biochar, requiring more time for physical and microbial break down for their beneficial impacts on plant growth^15,20^. Additionally, the biochar used in this study was pelleted with small additions of a binder, which helps maintain pellet integrity in the soil longer than usual. Similar results were reported in a study on corn, where increased availability of Ca and Mg by 77–320% contributed to higher nutrient uptake resulting in 28–140% enhanced corn yields in subsequent years^15^. In this study, higher plant population, LAI, number of open bolls and boll weight, were obtained as reported in the earlier studies^10,11,30^. The increased cotton crop growth and yield observed was likely due to the addition of nutrients like N, P, and K released by slow mineralization of biochar that the plant is able to use for longer periods during the growth phase of the crop. The release of nutrients to the growing cotton plants probably contributed to high lint and seed yields^10,11,30–32^^.^ It is pertinent to recall the N, P and K levels in the biochar used in this study are insufficient (Table 1) to meet major nutrient requirements for cotton growth. Potassium, however, typically concentrates in biochar and tends to be highly available. Cantrell et al. showed that total K (in combination with Na) concentration was an important predictor of biochar electrical conductivity, indicating that the form of K in biochar was water-soluble^33^. They found K availability ranging from 3.5 to 100 percent of the total K present. Biochars contain a plethora of inorganic elements, besides K, but the supply of available nutrients can be quite variable. Biochar function is not to be an exact replacement for inorganic fertilizer. Hence, the organic fertilizers were applied following the recommendations of Mississippi State University cotton extension.

Similarly, cotton lint quality was affected by biochar application during the three-year study, but the trends were not very clear. For example, micronaire, which is a measure of fiber fineness and maturity, and represents the surface area of lint, was significantly lower at 10 t ha^−1^ in 2019 and while 40 t ha^−1^ biochar applied plots recorded highest values in 2020 and 2021. It is not clear why micronaire was low in biochar applied plots at 20 t ha^−1^ compared to that of 10 t ha^−1^ application rate in 2020. It is pertinent to note here that the highly variable effect of biochar on crop yield is generally attributable to multiple factors ranging from application rate, depth of incorporation, feedstock used for biochar production, pyrolysis conditions, time for biochar to breakdown, soil type and fertility, management, and geographic location^21^. In this study, a larger variability across biochar treatments observed in the first year, is partly attributable to the slow rate of pellet breakdown in soil which lasted all harvest season. As biochar ages, it is slowly incorporated into soil aggregates^28^. This process involves physical breakdown of pellets with concomitant increased interaction of biochar and soil surfaces. The slow rate of breakdown was likely exacerbated with a lower precipitation rate in 2019, compared to the two remaining years.

The cotton industry needs longer and stronger cotton fibers. Only in the third year did the fiber strength increased proportionally with the biochar application rate, and biochar applied plots recorded significantly higher strength in the second and third years of study. Application of biochar in pellet form is advantageous in that it can be done with existing farming equipment and because of its much higher bulk density, transportation efficacy is significantly improved. Drop spreaders used for granular fertilizer application are an example. Bulk density of pelleted biochar is consistent from batch to batch and allows for higher precision of application than if it were in powder form. Pelleted biochar will also remain in the ground and therefore its application is not restricted to low windy conditions. Once in contact with soil, biochar is likely to undergo changes in physical–chemical properties dependent on the biochar, the soil, and the climatic conditions. With each hydration event and over time, biochar can fragment to smaller particles, and aggregate and interact with soil, forming soil-biochar aggregates. Interaction between biochar and soil is a complex set of events and entails biochar, soil, microbes and plant roots; being affected by soil moisture and temperature. These interactions start with mineral weathering processes such as hydrolysis, dissolution, carbonation and decarbonation, hydration, and redox reactions. The dissolution and leaching of soluble salts (e.g., K and Na carbonates and oxides) present in the biochar is the first reaction among all the interactions^34,35^. As the crop grows, there are also complex interactions between biochar with plant roots and microorganisms. Root hairs can penetrate water-filled macropores of the particle and the organic compounds (including low- and high-molecular-weight compounds such as free exudates and mucilage); sloughed-out cells and tissues; and lysates from the growing root can be absorbed by biochar surfaces^36^.

In this study, increased biochar application rates increased PWUE in the third year, following the enhanced yield response. Biochar persists in the soil for multiple years after its application. The greater persistence of biochar in soil originates mainly from the fact that the charring process results in changes in the properties of the material that confer greater persistence and longer residence times. A greater persistence of biochar in soil means that it continues for a longer period of time, with effects on the nutrient and water availability. Over time, surface oxidation processes on the biochar can lead to the development of cation retention. Properties of anthropogenic soils, specifically in Amazonia, locally referred to as Terra Preta de Indio are often seen as a proxy for long-term effects of biochar on soil productivity but have to be taken with some scrutiny due to their complex history of formation^37,38^. Attributes often claimed for biochars include the ability to retain plant fertilizers and to reduce the bioavailability of organic and inorganic contaminants. Ability of biochars to retain nutrients is attributed to its great porosity and quantity of functional groups. Similar to soils, biochar cation exchange capacity is developed upon exposure to oxygen and water, creating oxygenated surface functional groups^39,40^. Low temperature biochars retain more organic functional groups, so the potential exists for higher initial nutrient retention with these biochars. Nutrient retention may also be a function of short- and long-term oxidation once biochar is introduced into the soil^41^. For that reason, benefits of its application can be extended for multiple years after application. Kätterer et al. applied 50 t ha^−1^ to maize and soybean crops for two seasons and found that yields responded positively to pyrolyzed Acacia biochar and more importantly, the benefits of biochar application to soil such as increased soil porosity, pH, plant-available phosphorus and soil water-holding capacity, continued for over 10 years after application^42^. The same study reported an average grain yield advantage of 1.17 t ha^−1^ for maize and 0.43 t ha^−1^ for soybean, and overall effective addition of 28.1 t ha^−1^ C and 0.73 t ha^−1^ N. Biochar properties after application to soil, change with time as it continues to interact with microbes, soil organic and mineral matter, as well as plant roots^43,44^. Joseph et al. described three chronological reactions of applied biochar in soil: dissolution: from one to three weeks; reactive surface development from one to six months, and aging, beyond 6 months^28^. Wang et al. carried out a comprehensive meta-analysis study on biochar effects on the morphology and growth of plant roots^43^. Biochar amendment was found to increase root biomass by about 32% and root surface area, number of root tips, the number of N_2_-fixing nodules and specific root length by 39%, 17%, 25% and 52%, respectively. These changes in the presence of biochar were more pronounced in annual crops than in woody perennials and were significantly higher in legumes than in non-legumes.

This study revealed that the non-biochar treatment returned higher profits in all three years vis a vis biochar applied ones. This is reflected from the higher application rates of biochar leading to increased production costs, which could not be offset by the enhanced yields resulting from higher application rates. However, longer-term effects of biochar application on crop yield beyond the three years is expected to continue given the resilient nature of biochar^44,45^. Additionally, biochar might help mitigate situations of water and/or nutrient deficiencies. In addition to yield enhancement, biochar addition can be considered as an income/economic decision for growers as a “carbon credit” to sequester carbon and probably nitrogen credits in the future. A meta-analysis containing 437 comparisons between biochar treated soils and biochar non-treated soils showed that biochar treatment leads to a significant decrease of N_2_O emissions, between 33 and 45%^44,45^. The persistence of the carbon in biochar is correlated with the ratio of hydrogen to organic carbon (H/Corg). The biochar used in this study was produced via torrefaction, which is carried at low pyrolysis temperature. This biochar typically has a high H:Corg ratio (Table 1), therefore, it does not have the same desirable persistence in the soil as biochar produced at higher temperatures. In opposition, this biochar is also rich in volatile matter (Table 1), which contains water-soluble and mineralizable compounds. Low-temperature biochar typically contains higher concentrations of water-extractable organics. These lower volatility compounds such as organic acids, a dominant compound in biochars produced at lower pyrolysis temperature, have been shown to stimulate microbial activity and increase abundance^46,47^.

From another viewpoint, the current field was mostly grown for soybean under an intensive production system before this experiment, and therefore improvement in soil fertility levels per se, might not have had a greater role in improving nutrient uptake by cotton plants. Hence, future studies should consider evaluating the nutrient levels at the tissue level with reference to critical developmental stages to help our understanding of the exact mechanism of biochar-soil–plant interactions at the micro and macro levels. It is also worth mentioning that additional years beyond the three-year study, even without further biochar addition, could potentially result in continued crop yield benefits as the biochar continues to reside and age in the soil. In the United States, no national carbon market exists. Still, several voluntary and regulatory markets have emerged which allow for purchases of carbon offsets accrued from carbon sequestration and conservation practices. In the state of California currently, each carbon credit is trading at USD 29.07, while in the compliance markets in the European Union, it is trading at USD 86.27 (https://carboncredits.com/carbon-prices-today/).

## Conclusions

The third year of continuous application of biochar derived from sugarcane bagasse had a significant positive impact on cotton productivity, increased open bolls and boll weight, and enhanced lint and seed yields. Both HVI and AFIS cotton lint quality parameters were impacted significantly by higher rates of biochar application. Although the productivity levels were significantly high in the third year of study in the biochar-applied plots, the economic profits were lower compared to the no-biochar plot. Hence, successive years of biochar application, the cost of biochar, and the distance between biochar source and growers field could influence the producer’s decision to apply biochar. This practice would be attractive to farmers who, upon applying, can receive cost-share funds available through Environmental Quality Incentives Programs for using biochar to increase soil carbon. Producers, if paid for carbon credits, i.e., for sequestering carbon, would also be relevant when ecological and climate change mitigation impacts are assessed.

## Data Availability

The original contributions presented in the study are included in the article/supplementary material. Further inquiries can be directed to the corresponding author.
